# Successfully treated hemi-central retinal artery occlusion following cardiac catheterization; case report

**DOI:** 10.1016/j.amsu.2021.02.021

**Published:** 2021-02-23

**Authors:** Abdullah Al-kasasbeh, Omar Saleh, Rasheed Ibdah, Sukaina Rawashdeh, Khalid Ibrahim

**Affiliations:** a Division of cardiology, Department of Internal Medicine, Faculty of Medicine, Jordan University of Science and Technology, Irbid, Jordan; b Department of Ophthalmology, Faculty of Medicine, Jordan University of Science and Technology, Irbid, Jordan; c Division of Cardiac Surgery, Departmrent of General Surgery and Urology, Jordan University of Science and Technology, Irbid, Jordan

**Keywords:** Catheterization, Complication, Retinal artery, Embolization, Case report

## Abstract

**Introduction and importance:**

Here we report a case of a middle-aged man who complained of blurred vision in his left eye 1 h post cardiac catheterization and proved to have central retinal artery occlusion, a dangerous but potentially treatable sight-threatening complication of cardiac-catheterization. The patient was successfully treated through an Ophthalmological surgical intervention.

**Case presentation:**

A 49- year-old male patient admitted to the coronary care unit as a case of non-ST-elevation-myocardial infarction. The patient underwent cardiac catheterization and stenting of the right coronary artery. One hour later, he complained of blurred vision in his left eye.

**Clinical discussion:**

Ophthalmological examination showed an inferior visual field defect in the left eye. Fundus fluorescein angiography revealed that the patient had a hemi-central retinal artery occlusion, a rare complication of cardiac catheterization. A pars plana vitrectomy eye surgery was performed with an excellent result.

**Conclusion:**

This case highlights the importance of early recognition and treatment of central retinal artery occlusion post cardiac catheterization

## Highlights

• Rarely, central retinal occlusion can complicate cardiac catheterization.
• Meaningful spontaneous recovery in vision in cases of central retinal artery occlusion occurs in less than 10%.
• What is peculiar about our case is that prompt surgical intervention resulted in an unexpectedly excellent result.
• The case highlights the need for prompt diagnosis and referral of patients with visual symptoms post cardiac catheterization.

## 1 Introduction

Well known causes of central retinal artery occlusion include atherosclerosis, hypertension, diabetes mellitus, hematologic disease, vascular disease, trauma, inflammation, tumors, and iatrogenic causes [1,2]. Rarely, central retinal occlusion can complicate cardiac catheterization. Here, we report a case of successfully treated iatrogenic hemi-central retinal artery occlusion following cardiac catheterization.

## 2 Case presentation

A 49-year-old male patient hypertensive, smoker with positive family history of premature coronary artery disease who presented with heavy retro-Sternal chest pain.

**Fig. 1:**
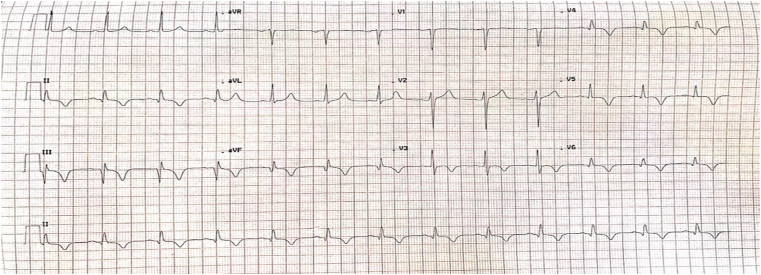
Electrocardiogram showing sinus rhythm with Q waves and T-wave inversion in lead II, III, and aVF.

At admission, the patient had no chest pain. Physical examination revealed a blood pressure of 130/85 mmHg and heart rate of 85/minute. Cardiac and respiratory examination were unremarkable and there was no carotid bruit. Electrocardiogram showed sinus rhythm with Q waves and T-wave inversion in lead II, III, and aVF (Fig. 1). Echocardiogram showed a left ventricular ejection fraction of 60% with no regional wall motion abnormalities, moreover, it showed normal structure and function of the cardiac valves. No intracardiac thrombi were found. Cardiac biomarkers were elevated.

**Fig. 2:**
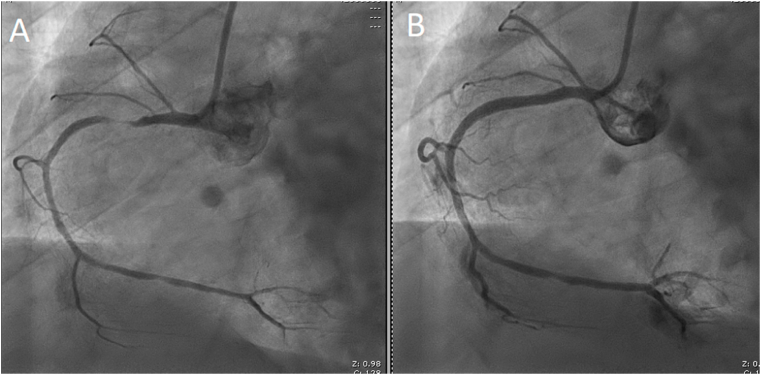
Coronary angiogram pre- and postpercutaneous coronary intervention (PCI). A, pre –PCI angiogram image showing severe stenosis of the right coronary artery. B, Post-PCI angiogram showing widely patent artery.

The patient underwent cardiac catheterization and was found to have one vessel disease with 90% stenosis of the right coronary artery. A drug-eluting stent was successfully implanted in the right coronary artery (Fig. 2).

**Fig. 3:**
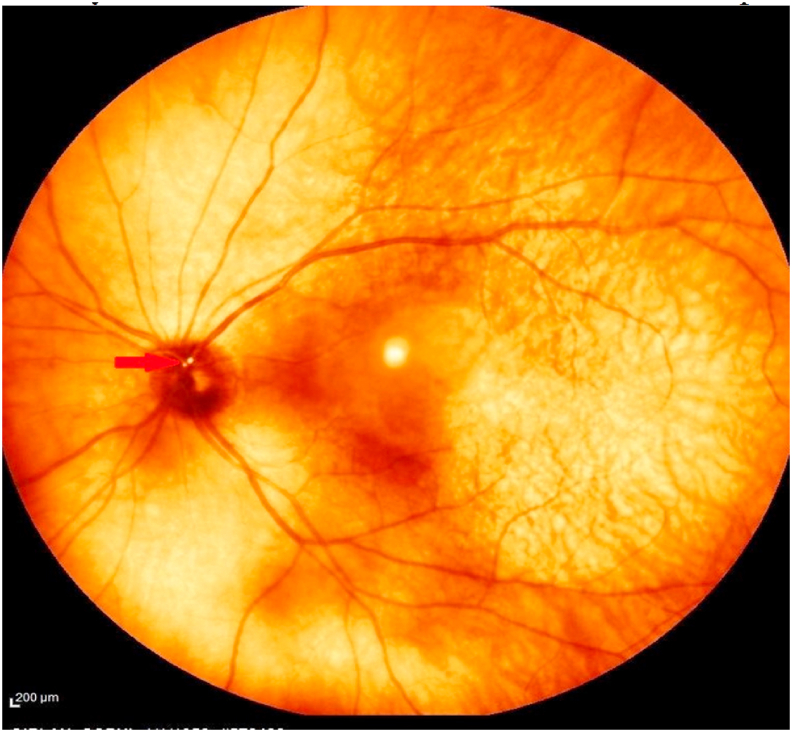
Fundus false-color infrared photograph of the left retina shows a small yellow round embolus at the origin of the superior retinal artery (red arrow). (For interpretation of the references to color in this figure legend, the reader is referred to the Web version of this article.)

One hour after intervention, the patient reported acute painless blurred vision in the left eye associated with a lower visual field defect. History from the patient revealed no prior visual complaints or amaurosis fugax. Ophthalmological examination of the left eye showed visual acuity of 6/60 with normal anterior segment and intraocular pressure. Visual field testing showed complete inferior visual field defect in left eye. Fundoscopic exam showed moderate macular edema and a small yellow arterial embolus at the level of the optic nerve head (Fig. 3).

**Fig. 4:**
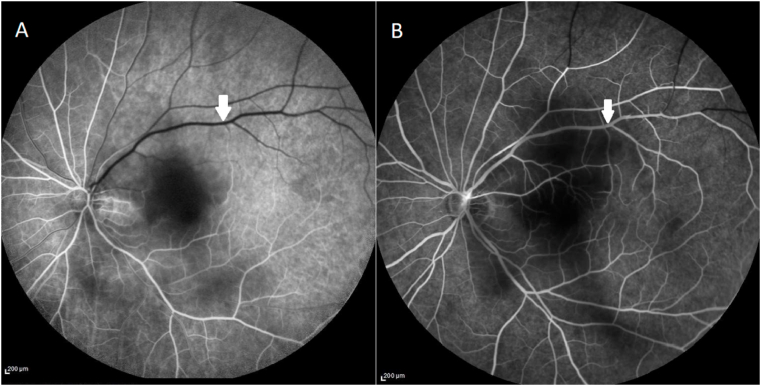
Fundus fluorescein angiograms at patient's presentation at 12 seconds (A) and 1 minute (B) after dye injection showing a delay of arterial filling in the superior retinal territory consistent with superotemporal retinal artery occlusion (white arrows).

Fundus fluorescein angiography (Fig. 4) revealed a 7-s filling delay in the superior-temporal retinal artery; findings consistent with hemi-central retinal artery occlusion.

**Fig. 5:**
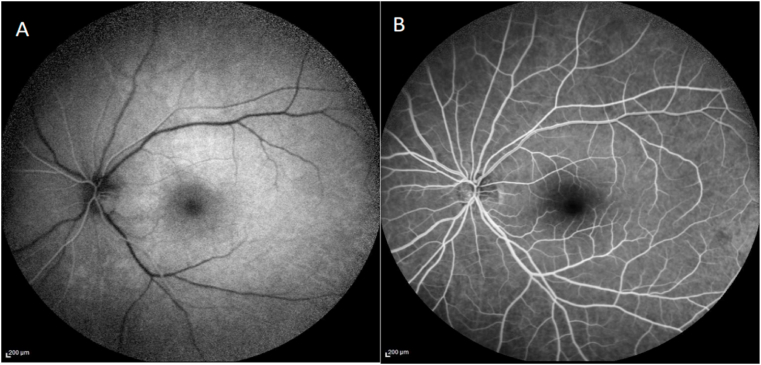
Fundus fluorescein angiograms at 5 seconds (A) and 22 seconds (B) after dye injection showing restoration of normal arterial filling with no filling delay.

A pars plana vitrectomy eye surgery under local anesthesia was performed. Massaging of the superior retinal artery wall over the embolus using a silicone-tipped aspiration cannula was performed in conjunction with induced ocular hypotony, which resulted in dislodgment of the embolus and migration into the peripheral circulation. The patient noted an immediate marked improvement in his vision. Four weeks later, the patient had a visual acuity of 20/25 and normal visual fields. There was complete resolution of the embolus and the artery occlusion and restoration of retinal blood flow (Fig. 5).

The study was approved by the Institute Research Board at King Abdullah University Hospital and Jordan University of Science and Technology. The work has been reported in line with the SCARE 2020 criteria [3]. Written informed consent was obtained from the patient for publication of this case report and accompanying images. A copy of the written consent is available for review by the Editor-in-Chief of this journal on request.

## 3 Discussion

Retinal artery occlusion is an uncommon sight-threatening condition, with an incidence of about 1/100,000 in the general population and usually presents with a sudden painless severe loss of vision or a visual field defect in the affected eye. The central retinal artery is most commonly occluded by a cholesterol embolus (Hollen-horst plaque) which usually originates from atherosclerotic plaques in the carotid arteries, aortic arch, or ophthalmic artery with an average age of 62 years at presentation. Embolic occlusions that affect younger people, however, are commonly cardiac in origin and emboli may arise from calcific heart valves, atheromas, or myxomas in cardiac chambers (**4).**

In-situ thrombosis may also cause retinal artery occlusion. Thrombi may be caused by atherosclerotic disease or may be due to collagen-vascular disease, as in systemic lupus erythematosus, inflammatory conditions, as in giant cell (temporal) arteritis, polyarteritis nodosa, Behcet disease, and syphilis, or hypercoagulable states such as polycythemia vera, sickle cell anemia, multiple myeloma, factor V Leiden, and antiphospholipid syndrome. Rapid differentiation between thrombo-embolic versus arteritic central retinal artery occlusion is important as optimal therapy differs and the rapid administration of steroids for vasculitic causes is associated with improved outcomes [5]. The rate of retinal emboli after cardiac catheterization is still controversial. In one study, it approaches 6.3%. Most of the time those emboli are clinically silent and do not produce any visual sequelae. [6,7].

During cardiac catheterization the central retinal artery is most commonly occluded by a cholesterol embolus which are thought to be caused by the disruption of a vascular plaque usually in the aortic arch (mainly by catheter manipulation) with the release of subendothelial cholesterol crystals into the blood stream [4,8].

Cerebral blood flow as well as the diameters of the cerebral and the central retinal blood vessel are thought to play major role in determining the destination of the embolus. Whereas large atheromas are big enough to lodge in the small retinal artery, very small one cannot occlude it [1].

Meaningful spontaneous recovery in vision in cases of central retinal artery occlusion occurs in less than 10%.

Treatment for embolic central retinal artery occlusion is typically conservative and includes ocular massage, ocular anterior-chamber paracentesis, sublingual isosorbide dinitrate, intravenous acetazolamide, intravenous mannitol or oral glycerol, hyperbaric oxygen, and isovolumic hemodilution. None of these treatments have proven effective and their use is largely based on anecdotal reports and small case series [9].

In addition, some of these interventions such as intra-arterial thrombolysis and intraocular surgery may carry high rates of complications and are usually not adopted by most ophthalmologists [[10], [11], [12]].

The utilization of vitrectomy has been reported with variable success rates and visual benefit and a potential for complications. Surgical techniques included stylet cannulation of the central retinal artery, intra-operative controlled hypotony, and retinal wall massage [[13], [14], [15]].

## 4 Conclusion

What is peculiar about our case is that prompt surgical intervention by the ophthalmology service resulted in an unexpectedly excellent result with complete restoration of blood flow in the retinal arteries and almost complete visual recovery.

This case highlights the need for prompt recognition and urgent referral of any patient with visual symptoms post cardiac catheterization.

## Provenance and peer review

Not commissioned, externally peer-reviewed.

## Declaration of competing interest

All of the authors declare that they have no conflict of interests.

## Registration of research studies

N/A.
